# Outbreak of Alternaria Black Spot of Pomegranate (*Punica granatum* L.) in Italy as a Consequence of Unusual Climatic Conditions

**DOI:** 10.3390/plants13142007

**Published:** 2024-07-22

**Authors:** Gaetano Tirrò, Sebastiano Conti Taguali, Antonella Pane, Mario Riolo, David Ezra, Santa Olga Cacciola

**Affiliations:** 1 Department of Agriculture, Food and Environment, University of Catania, Via S. Sofia 100, 95123 Catania, Italy; gaetano_tirro@yahoo.it (G.T.); sebastiano.contitaguali@unirc.it (S.C.T.); apane@unict.it (A.P.); olga.cacciola@unict.it (S.O.C.); 2 Department of Agricultural Science, University Mediterranea of Reggio Calabria, 89122 Reggio Calabria, Italy; 3 Department of Plant Pathology and Weed Research, Agricultural Research Organization, ARO, The Volcani Institute, P.O. Box 15159, Rishon LeZion 7528809, Israel; dezra@volcani.agri.gov.il

**Keywords:** pomegranate, black spot disease, climate change, barcode, *Alternaria* spp.

## Abstract

Alternaria black spot of pomegranate (*Punica granatum*) was reported for the first time in Italy. In spring 2023, an outbreak of this disease was noticed in commercial pomegranate ‘Wonderful’ orchards of the municipality of Misterbianco (Sicily), following an unusually rainy period. A total of 30 randomly selected *Alternaria* isolates recovered from typical necrotic spots of leaves and fruits were characterized. Based on the colony morphology on solid agar media (PDA and MEA), isolates were separated into three distinct morphotypes (1, 2, and 3). The first two morphotypes comprised only isolates from fruits, while morphotype 3 comprised only isolates from leaves. Multigene phylogenetic analysis of four DNA regions, including internal transcribed spacer (ITS), translation elongation factor 1-α (EF-1α), glyceraldehyde-3-phosphate dehydrogenase (GAPDH), and a SCAR marker (OPA10–2), identified the isolates of morphotypes 1 and 2 as *Alternaria alternata* and morphotype 3 isolates as *A. arborescens*. In pathogenicity tests on unwounded leaves and fruit, the isolates of all three morphotypes produced symptoms on the leaves of three pomegranate cultivars, ‘Acco’, ‘Wonderful’, and ‘Etna’. The symptoms on ‘Acco’ leaves were the least severe. Conversely, the fruits of ‘Acco’ were the most susceptible. The isolates of morphotypes 2 and 3 were not pathogenic on the fruits of ‘Wonderful’ and ‘Etna’. This is the first report of Alternaria black spot in Italy and of *A. arborescens* associated with Alternaria black spot of pomegranate worldwide.

## 1. Introduction

Pomegranate (*Punica granatum* L., *Punicaceae* family) is gaining renewed interest as a fruit crop in different geographic areas of the world [1,2]. Pomegranate fruit is known for both its flavor and beneficial nutritional properties [3]. Moreover, pomegranate fruit peel extracts, being rich in polyphenolic compounds with antioxidant and antimicrobial activity, are being valued as drugs, natural pesticides, and food preservatives [4,5,6,7]. In Italy, the cultivation of this deciduous fruit crop has been rapidly expanding, and from less than 10 ha in 2008, it is now estimated to be over 1500 ha [2,3,4,8]. The major pomegranate producing regions are Apulia and Sicily (southern Italy), but new plantings are also being established in regions of central and northern Italy [9]. Imported cultivars are replacing traditional local selections for their higher productivity and market quality. The most popular cultivars imported from abroad include, among others, ‘Wonderful’, whose origin as a cultivar is controversial (USA/Israel) and which has become the standard type; ‘Mollar de Elche’ and ‘Parfianka’, both originating from Spain; and ‘Acco’, of Israeli origin. As a consequence of the crop expansion in new areas, the intensification of cropping systems, and the introduction of new pomegranate genotypes, new and rare diseases have emerged [10,11]. Pre- and post-harvest pomegranate diseases have been reported from around the world (as reviewed by Bregant et al. [9]; [12,13,14]). Common pathogens affecting pomegranate include fungi, such as *Botrytis cinerea*, *Alternaria alternata*, *Penicillium implicatum*, *Coniella granati*, *Cercospora punicae*, *Botryosphaeria dothidea*, and *Aspergillus niger*, as well as bacteria, such as *Xanthomonas axanopodis* pv. *punicae*, which causes bacterial blight. These pathogens lead to significant financial, nutritional, and post-harvest losses along the value chain [11,13].

Between the end of May and the beginning of June 2023, an outbreak of a disease new to Italy was noticed in a pomegranate-growing area in the municipality of Misterbianco (Catania province, Sicily, southern Italy). The symptoms on leaves and fruits were very similar to those of Alternaria black spot of pomegranate reported in other Mediterranean countries [15,16]. The disease appeared following a rainy period that was unusual for this season (with a total precipitation in April and May of 89.6 mm, as registered by the Sicilian Agrometeorological Information Service (SIAS)) and was first detected in a farm that was the first in the area to introduce new pomegranate cultivars and cropping systems. Moreover, the farm also hosts a collection of diverse commercial pomegranate cultivars and genotypes, including local selections. The objectives of this study were (i) to determine the etiology of the disease; (ii) to characterize the causal agent(s); and (iii) to evaluate the disease severity in selected commercially popular pomegranate varieties.

## 2. Results

### 2.1. Disease Symptoms and Isolates

**Figure 1:**
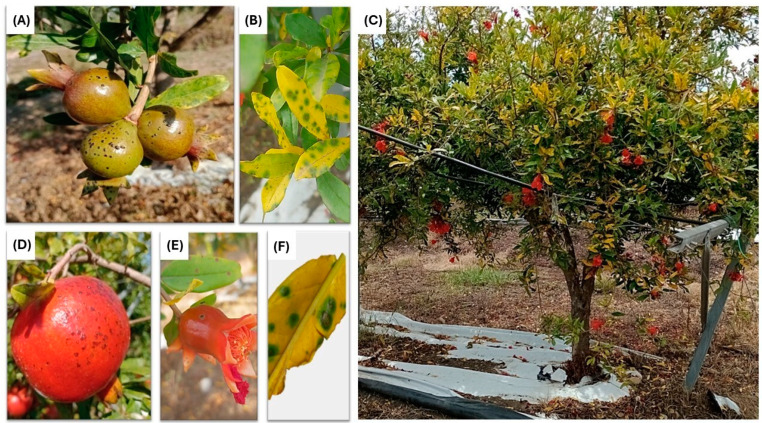
Black and brown spots on fruits, leaves, and flowers of pomegranate. (**A**) Numerous necrotic spots on fruits; spots become crusty and occasionally coalesce. (**B**) Pinpoint necrotic spots on young pomegranate leaves. (**C**) Defoliation of twigs and severe chlorosis of pomegranate leaves and black spots on their surfaces. (**D**) External symptoms of black spots on fruit. (**E**) Black spot symptoms on pomegranate flower (**F**). Close-up image of necrotic spots on leaf.

The symptoms of the disease were small, black to dark brown spots of up to 4 mm in diameter, scattered on the leaf blades, flowers, and fruit peel (Figure 1A–D). On green leaves, spots were often surrounded by a pale chlorotic halo. Severely affected leaves became entirely chlorotic, and spots stood out on the chrome yellow background of the leaf blades as they were surrounded by a green halo (Figure 1C). A close-up of the leaf spots revealed that they tended to enlarge and were zonate, forming concentric necrotic rings. Infected leaves dropped with the consequent defoliation and dieback of twigs. In many cases, symptoms were uniformly distributed across the entire tree canopy (Figure 1B), but typically, single shoots or only part of the canopy stood out due to severe leaf chlorosis and defoliation. On fruits, spots were like crusts and occasionally merged with each other (Figure 1A,D), they were superficial, and the necrosis was confined to the rind surface. The disease severity varied from a single spot per leaf or fruit to numerous spots covering the entire surface of the leaf blade or fruit, including the calyx. The disease was initially detected in an orchard spanning approximately 2 hectares, which included various cultivars, such as ‘Wonderful’ (the predominant variety), ‘Acco’, ‘Mollar de Elche’, ‘Parfianka’, and two local selections named ‘Etna’ and ‘Primosole’. The symptoms were mainly observed on ‘Wonderful’, affecting over 60% of the trees. The other cultivars showed no symptoms of the disease. The ‘Etna’, ‘Primosole’, and ‘Parfianka’ cultivars were planted at the top of a hill in terraced soil, ‘Wonderful’ was located in the valley bottom, and ‘Acco’ and ‘Mollar de Elche’ were planted on a slope at an intermediate altitude. The ‘Acco’ and ‘Mollar de Elche’ plot was more than 200 m away from the ‘Wonderful’ plot, with a river separating them. As summer progressed, all symptomatic leaves abscised, and by the harvest time in October, only a few fruits (less than 2%) exhibited symptoms on the rind. Isolates from symptomatic leaves and fruits collected in June consistently yielded *Alternaria* isolates.

### 2.2. Morphological Characterization of Isolates

**Figure 2:**
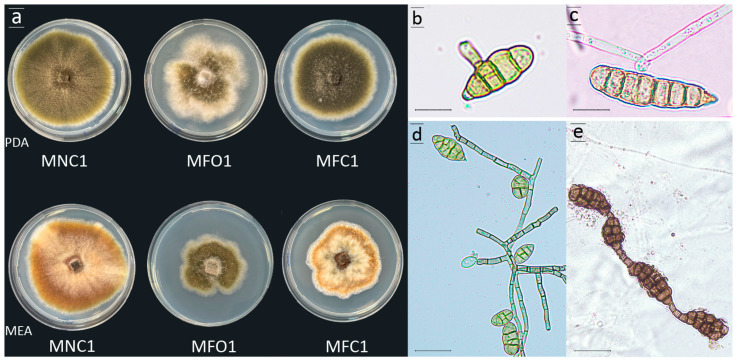
(**a**) Growth patterns and colony morphologies of isolates of *Alternaria* spp. recovered from pomegranate fruits and leaves with symptoms of Alternaria black spot collected in Sicily. From left to right: MNC1, MFO1, and MFC1 isolates, each representing a distinct morphotype (morphotype 1, 2, and 3, respectively), grown on PDA (top) and MEA (bottom), and incubated at 25 ± 1 °C for 7 days in the dark; (**b**,**c**) microscope images of germinating conidia of *Alternaria alternata* (MNC1 and MFO1) and *A. arborescens* (MFC1), respectively; and (**d**,**e**) septate conidia and hyphae of MNC1 and MFC1 isolates, respectively.

*Alternaria* isolates produced dark, brown, club-shaped catenulate conidia, with longitudinal (0 to 5) and transverse (3 to 6) septa and a prominent beak, produced on either simple and short or long, occasionally branched, conidiophores (Figure 2d,e). The conidia measured (12−) 20.1 (−28) × (6−) 8.9 (11) µm (Figure 2b,c). The colony morphology varied, and isolates were grouped into three distinct morphotypes based on the colony morphologies on potato dextrose agar (PDA) and malt extract agar (MEA) (Figure 2a). Colonies of morphotype 1 isolates were appressed, velvety, slightly radiate, olivaceous on PDA, and light brown on MEA, with distinct margins on both media. Colonies of morphotype 2 isolates grew more slowly than colonies of the two other morphotypes, and they were olivaceous in the center, with white aerial mycelium in the periphery and very irregular margins on both PDA and MEA. Colonies of morphotype 3 isolates were uniform, velvety, and dark green–olivaceous, with an appressed mycelium and regular margins on PDA and slightly petaloid, light brown, with irregular margins on MEA (Figure 2a). Overall, 30 randomly selected isolates (10 per morphotype) were selected for further characterization (Table 1).

### 2.3. Molecular Characterization

**Figure 3:**
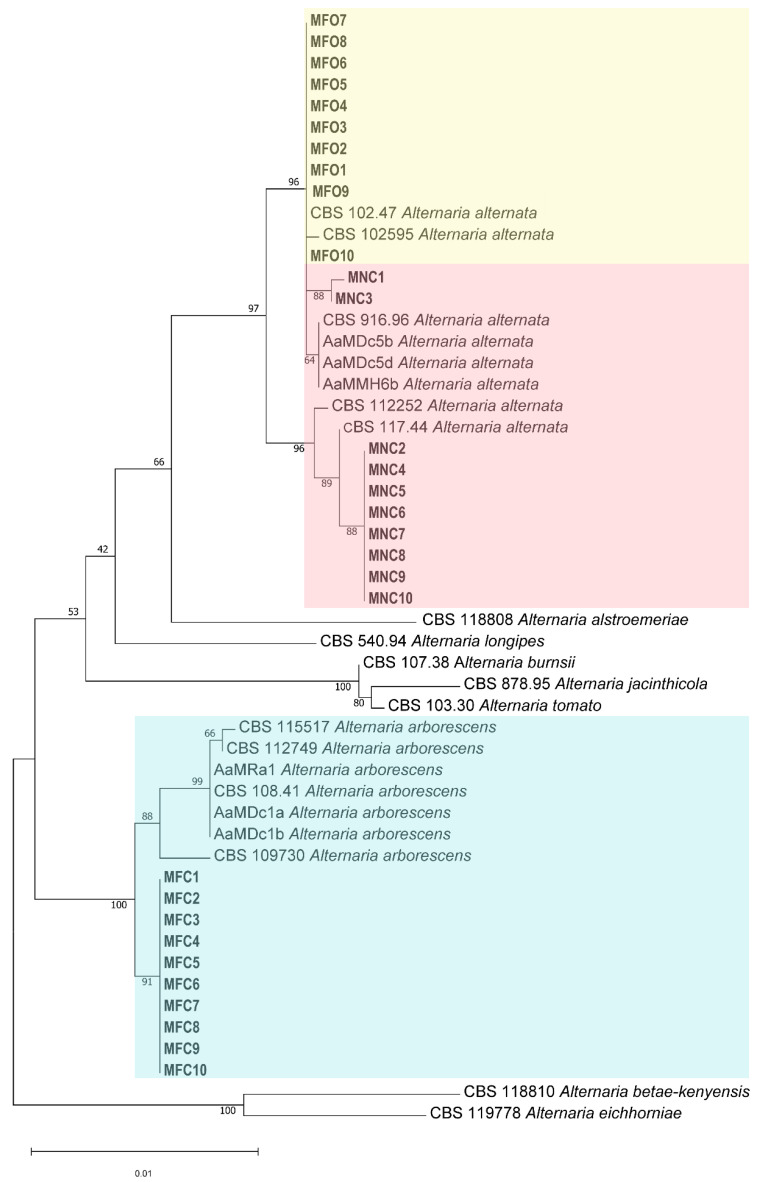
Internal transcribed spacer (ITS), glyceraldehyde-3-phosphate dehydrogenase (GAPDH), translation elongation factor 1-α (EF-1α), and one SCAR marker (OPA 10-2) multilocus phylogenetic tree developed using the maximum-likelihood method, based on the Tamura–Nei model. The tree with the greatest log likelihood (−3746.14) is shown. The relationships between the 30 isolates from leaves and fruits of pomegranate sourced from southern Italy and the reference isolates of *Alternaria alternata*, *A. arborescens*, and other *Alternaria* spp. Two species, *A. betae-kenyensis* and *A. eichhomiae,* were used as the outgroup of the phylogenetic tree.

The *Alternaria* isolates obtained in this study (Table 1) and the reference isolates (Table 2) were grouped based on their four-gene phylogeny, including ITS, GAPDH, EF-1α, and OPA 10-2 sequences [4,17]. According to the phylogenetic analysis, 20 out of the 30 isolates were associated with *A. alternata* and clustered with reference isolates of *A. alternata*, including the isolates CBS 916.96 and CBS 112252 (Figure 3). Ten isolates were clustered within the *A. arborescens* species complex along with the strains CBS 109730 from *Solanum lycopersicum*, CBS 105.24 from *S. tuberosum*, CBS 108.41 from wood, CBS 112749 from *Malus domestica*, CBS 118389 from *Pyrus pyrifolia*, and CBS 115517 from *M. domestica*. The multigene-phylogenetic analysis of those four DNA regions identified morphotypes 1 and 2 isolates as *A. alternata* and morphotype 3 isolates as *A. arborescens*.

### 2.4. Pathogenicity Test

**Figure 4:**
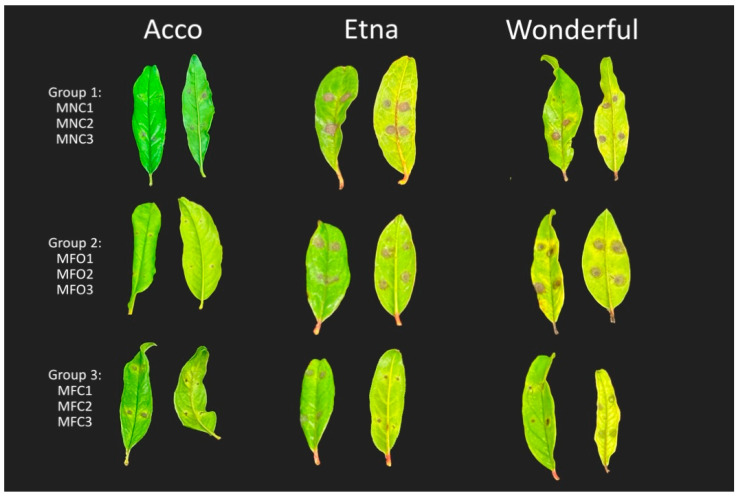
Differential susceptibility of leaves of pomegranate cultivars Acco, Etna, and Wonderful to infection by nine *Alternaria* isolates of the three diverse morphotypes (morphotypes 1, 2, and 3, indicated as groups 1, 2, and 3, respectively). In detail, group 1: MNC1, MNC2, and MNC3; group 2: MFO1, MFO2, and MFO3; and group 3: MFC1, MFC2, and MFC3. Necrotic spots with a chlorotic halo on fully expanded young leaves inoculated with a conidial suspension (10^6^ conidia mL^−1^) without wounding, three days after inoculation (dpi).

**Figure 5:**
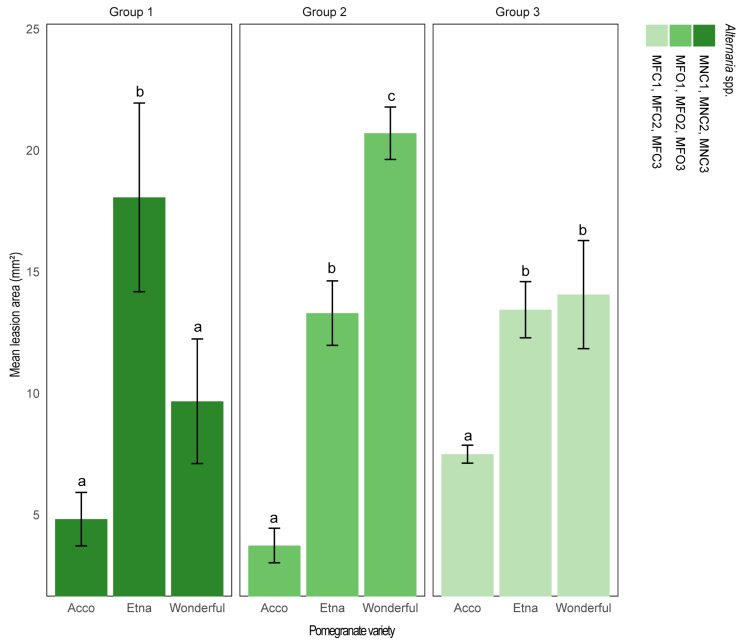
Differential susceptibility of leaves of pomegranate cultivars Acco, Etna, and Wonderful to infection by nine *Alternaria* isolates of three diverse morphotypes (morphotypes 1, 2, and 3, indicated as groups 1, 2, and 3, respectively). Mean area (±SD) of necrotic lesions (mm^2^) induced by each group of isolates on fully expanded young leaves inoculated with a conidial suspension (10^6^ conidia mL ^−1^), without wounding, three dpi. Within each group of isolates, values sharing the same letters are not statistically different according to Tukey’s honestly significant difference (HSD) test (*p* ≤ 0.05).

All *Alternaria* isolates tested produced necrotic circular lesions on the leaves of the three pomegranate cultivars tested (Figure 4). Significant differences were observed in the symptom severity between the three pomegranate cultivars. In detail, ‘Etna’ showed severe symptoms, with isolates of all three morphotypes; ‘Wonderful’ showed very severe symptoms with isolates of morphotypes 2 and 3 but less severe symptoms with morphotype 1 isolates; and ‘Acco’ showed mild symptoms with isolates of all three morphotypes (Figure 5). No symptoms were observed in control leaves, and the same fungi used for artificial inoculations were re-isolated from necrotic lesions of symptomatic leaves.

**Figure 6:**
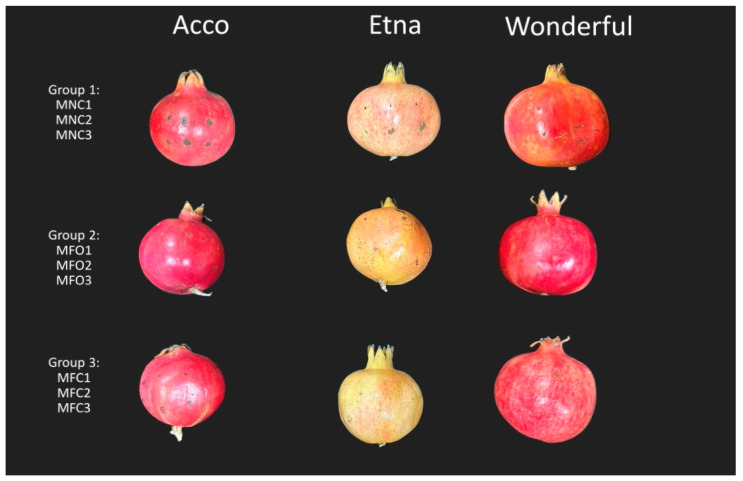
Differential susceptibility of fruits of pomegranate cultivars Acco, Etna, and Wonderful to infection by nine *Alternaria* isolates of three diverse morphotypes (morphotypes 1, 2, and 3, indicated as groups 1, 2, and 3, respectively). Necrotic spots on the peels of ripe fruits inoculated with a conidial suspension (10^6^ conidia mL ^−1^) without wounding, 10 days after inoculation (dpi).

**Figure 7:**
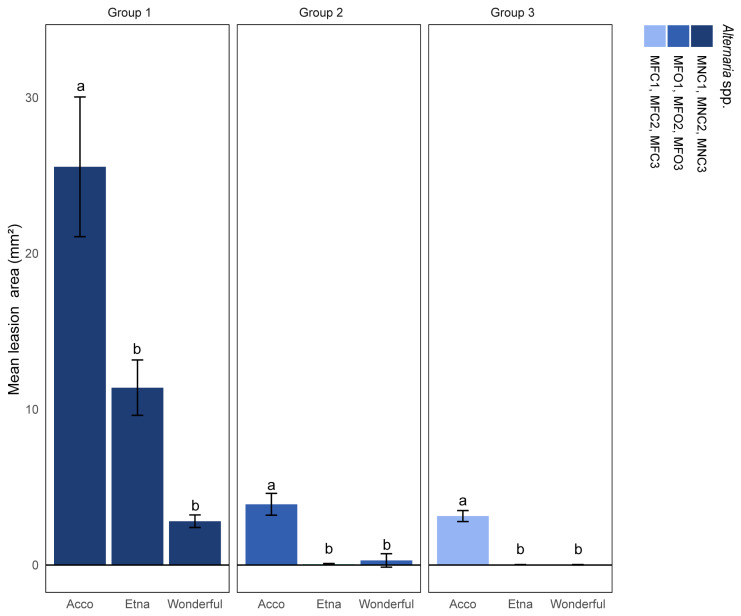
Differential susceptibility of ripe fruits of pomegranate cultivars Acco, Etna, and Wonderful to infection by nine *Alternaria* isolates of three diverse morphotypes (morphotypes 1, 2, and 3, indicated as groups 1, 2, and 3, respectively). Mean area (±SD) of necrotic lesions (mm^2^) induced by each group of isolates on pomegranate fruits inoculated with a conidial suspension (10^6^ conidia mL ^−1^), without wounding, 10 dpi. Within each group of isolates, values sharing the same letters are not statistically different according to Tukey’s honestly significant difference (HSD) test (*p* ≤ 0.05).

In pathogenicity tests on fruits, only morphotype 1 isolates (MNC1, MNC2, and MNC3), identified as *A. alternata*, produced noticeable necrotic lesions on the fruit peels of all three pomegranate cultivars (Figure 6 and Figure 7). The lesion size was significantly higher on ‘Acco’ fruits than on the fruits of ‘Wonderful’ and ‘Etna’. No significant difference was observed between the lesion sizes on the peels of ‘Wonderful’ and ‘Etna’ fruits. Isolates of morphotypes 2 and 3, identified as *A. alternata* and *A. arborescens*, respectively, produced noticeable necrotic lesions only on the peels of ‘Acco’ fruits, confirming this cultivar was the most susceptible to fruit infection among those tested. No symptoms were observed on the control fruits, and the same fungi used for the artificial inoculations were re-isolated from the necrotic lesions of symptomatic fruits.

## 3. Discussion

The *Alternaria* species responsible for the outbreak of Alternaria black spot of pomegranate in Sicily were identified as *A. alternata* and *A. arborescens*, on the basis of multigene phylogenetic analysis and according to the taxonomic criteria proposed by Woudenberg et al. [18]. In the Mediterranean region, Alternaria black spot of pomegranate was reported for the first time in Israel [16] and, a few years later, in Spain [15]. The disease was also reported in China, the United States, and, repeatedly, in India [13,19,20,21]. In Israel and Spain, the causal agent was identified as *A. alternata* [15,16,22]. This is the first report of Alternaria black spot in Italy and of *A. arborescens* associated with Alternaria black spot of pomegranate worldwide. Traditionally the taxonomy of *Alternaria* is controversial [17]. *A. alternata* and *A. arborescens* were regarded as separate species by Woudenberg et al. [18], but were assigned the rank of subspecies belonging to the same species complex by Armitage et al. [23]. However, both classification systems agree in recognizing that these two taxa are distinct lineages. Both *A. alternata* and *A. arborescens* were identified as causal agents of another disease of pomegranate fruit known as ‘heart rot’ or ‘black heart’, already reported in many Mediterranean countries, including Italy, as well as in India and the United States [4,8,24,25,26,27,28,29,30]. However, according to Ezra et al. [16] and Gat et al. [22], isolates causing black spots, although morphologically indistinguishable, are specific and distinct from those associated with heart rot. The present study revealed a great variability in both the morphology and pathogenicity of *Alternaria* isolates recovered from pomegranate leaves and fruits with symptoms of black spot in Sicily. On the basis of the colony morphologies, three distinct morphotypes were identified. One (morphotype 3) exclusively comprised isolates recovered from leaves and was identified as *A. arborescens*, while the other two morphotypes (morphotypes 1 and 2) exclusively comprised isolates recovered from fruits and were identified as *A. alternata* on the basis of phylogenetic analysis. These findings suggest differences in the ecology of these two closely related but genetically distinct *Alternaria* species. Moreover, isolates of morphotypes 1 and 2 clustered into two separate subclades of the multilocus phylogenetic tree, indicating that the *A. alternata* morphotypes were also genetically distinct. All isolates, irrespective of the *Alternaria* species and morphotype, were pathogenic on detached unwounded leaves. However, remarkable differences in susceptibility to leaf infections were observed among the three pomegranate cultivars tested. In the pathogenicity tests on leaves, ‘Acco’ was less susceptible than ‘Wonderful’ and ‘Etna’ to all *Alternaria* isolates. In contrast, in the pathogenicity tests on fruits, ‘Acco’ was more susceptible than the other two cultivars and was the only cultivar to be infected by all *Alternaria* isolates. A possible explanation is that fruits of ‘Acco’, an early-ripening cultivar, were more susceptible to infection by a necrotrophic pathogen like *Alternaria* because they were in a more advanced maturity phase. An alternative hypothesis is that the discrepancy between the results of the pathogenicity tests on leaves and fruits depended on a differential organ susceptibility. Interestingly, only isolates of morphotype 1 (homologous isolates) were virulent on fruit, while isolates of morphotypes 2 (homologous isolates) and 3 (heterologous isolates) were only weakly pathogenic on fruits of ‘Acco’ and non-pathogenic on fruits of ‘Wonderful’ and ‘Etna’, once again suggesting an isolate specificity. All these aspects, including the disease susceptibility level of diverse pomegranate cultivars, the most appropriate test to determine this susceptibility, as well as the variability in pathogenicity between *Alternaria* isolates and their specificity, deserve to be further investigated as they have practical implications for cultivar selection, breeding programs, and disease management strategies. A more robust, consistent, and stable taxonomy of *Alternaria* would certainly be useful to this end. In general, the selection of resistant cultivars is an effective and more sustainable alternative to synthetic fungicides to manage pomegranate diseases [31], also in view of the very limited number of pesticides authorized for this crop in the EU.

Beyond Alternaria black spot, pomegranate production is challenged by other significant diseases that impact yield and fruit quality. Heart rot, caused by *Alternaria* spp., is a major concern, alongside various fungal, bacterial, and viral pathogens. Notable diseases include Cercospora leaf and fruit spot (*Cercospora punicae*), Botryosphaeria blight and fruit rot (*Botryosphaeria dothidea*), and bacterial blight (*Xanthomonas axonopodis* pv. *punicae*). Viral diseases, though less common, such as pomegranate mosaic virus and pomegranate yellow mosaic virus, also pose challenges [13,32,33]. While *Alternaria* spp. are significant leaf pathogens, they are not the only ones affecting pomegranates. *Cercospora punicae* and *Botryosphaeria dothidea* also contribute to leaf and fruit diseases, impacting overall plant health and productivity [32,33].

The outbreak of Alternaria black spot of pomegranate in late spring was favored by warm temperatures and the presence of young susceptible leaves. However, it can be speculated that it was a direct consequence of exceptionally heavy rains, since, normally in Sicily at this time of the year, the absence of rains is a limiting factor for the occurrence of epidemics of fungal leaf disease. In this respect, it can be expected that in coming years, the probability of epidemic outbreaks of this disease occurring in spring will increase as a consequence of climate change. As climate change continues to affect environmental conditions, causing increased temperatures and changes in rainfall patterns, the risk of disease outbreaks may increase [34,35]. These alterations can create favorable conditions for the proliferation of pathogens like *Alternaria* spp. Increased moisture is critical for the germination and spread of fungal spores [36]. The unusually wet period that occurred before the outbreak likely facilitated the development and severity of the disease. In addition, future climate scenarios predict more intense and frequent rainfall events, which could lead to more common and severe outbreaks of diseases that thrive in wet conditions [37].

However, in Sicily, and more generally in the Mediterranean region, climatic conditions in summer are not conducive to Alternaria black spot of pomegranate, which consequently, in this area, can be regarded as a minor disease or an occasional constraint. An exception could be pomegranate orchards in northern and central regions of Italy or planted in valley floors along the riverbanks. Moreover, this disease may be a serious constraint in humid subtropical or tropical areas with warm temperatures, such as Florida (USA) and some regions of China where the crop is expanding, as it may cause severe defoliation and yield losses in the form of leaf spot [21].

The severity and progression of Alternaria black spot of pomegranate in Sicily, as influenced by climatic conditions, are consistent with findings on Alternaria leaf spot of soybean, where environmental factors such as temperature and relative humidity significantly affect disease development. Fagodiya et al. [38] highlight that the optimal temperature ranges and relative humidity levels that promote disease progression in soybean for Alternaria leaf spot underscore the importance of similar climatic conditions in the epidemiology of Alternaria black spot in pomegranates. Rising temperatures can expand the geographical range of both pathogens and their hosts, and warmer winters might not be cold enough to limit the survival of certain pathogens, allowing them to persist year-round [37,39]. As climate change continues to alter environmental conditions, leading to warmer temperatures and changes in precipitation patterns, the risk of disease outbreaks may increase. Therefore, understanding the interaction between climatic variables and disease development is crucial for anticipating and managing future outbreaks in the context of a changing climate [38,40,41].

## 4. Materials and Methods

### 4.1. Fruit and Leaf Sampling and Alternaria Isolation

From May to November 2023, surveys were carried out in the commercial pomegranate orchard where the disease outbreak was first noticed. The orchard was in the municipality of Misterbianco (the Province of Catania, Sicily, Italy; 37.463109312245535, 14.971325900520004). The trees were 10 years old, spaced 3.5 × 6 m, and trained into a transversal Y system. A drip-irrigation system (self-compensating double-drip wing) and a plastic mulching sheet along the rows were used. From the end of May to the end of June 2023, symptomatic leaves and fruits of the pomegranate cultivar ‘Wonderful’ were sampled. Other varieties present in the orchards, such as Acco, Parfianka, Mollar de Alche, Primosole, and Etna, which were located in another area of the orchard (400 m away), did not exhibit symptoms.

Small tissue pieces (2–3 mm) were excised from necrotic spots on leaves and fruit peels, surface-disinfected by immersion in 1% NaClO for 1–3 min, rinsed twice in sterile distilled water (SDW), blotted dry in sterile conditions on sterile filter paper, and plated in Petri dishes on potato dextrose agar (PDA, Oxoid Ltd., Basingstoke, UK) amended with streptomycin sulfate (250 µg/mL) (Sigma-Aldrich, St. Louis, MA, USA). The dishes were incubated for seven days at 22 ± 2 °C in darkness. Pure *Alternaria* isolates were obtained by single-hypha subcultures on PDA. The isolates were preserved in the culture collection of the Laboratory of Molecular Plant Pathology of the Department of Agriculture, Food, and Environment (Di3A) at the University of Catania.

### 4.2. Morphological Characterization of Isolates

The isolates were grown in Petri dishes on PDA and malt extract agar (MEA; Sigma-Aldrich, Burlington, MA, USA). The dishes were incubated for 7 days at 25 ± 1 °C in darkness. The morphological characteristics (color, margin, diameter, and texture) of the colonies and their microscopic features (conidium and conidiophore branch morphology) were examined according to Aloi et al. [4].

### 4.3. Molecular Characterization of Isolates

The *Alternaria* isolates recovered from symptomatic leaves and fruits of pomegranate were grown on PDA for 7 days at 25 ± 1 °C. Then, mycelium from each isolate was harvested using a sterile scalpel, and genomic DNA was extracted using a PowerPlant^®^ Pro DNA isolation Kit (MO BIO Laboratories, Inc., Carlsbad, CA, USA), following the manufacturer’s protocol. The DNA was then stored at −20 °C. To characterize and determine the phylogenetic allocation of the 30 isolates from pomegranate fruits and leaves, a multilocus approach was adopted. Sequencing was performed on segments of four *Alternaria* barcoding genes/regions: internal transcribed spacer (ITS), translation elongation factor 1-α (EF-1α), glyceraldehyde-3-phosphate dehydrogenase (GAPDH), and a SCAR marker (OPA10–2). The primers used in this study for amplifying these genes/regions were as follows: ITS1/ITS4 for the ITS region [42], EF1-728F/EF1-986R for the EF-1α gene [43], GPD1/GPD2 for the GAPDH gene [44], and OPA10-2R/OPA10-2L for the OPA10-2 marker [4,17].

GeneAmp PCR System 9700 (Applied Biosystems, Monza-Brianza, Italy) was used for the PCR amplifications. Taq DNA polymerase recombinant (Invitrogen™) was used for all PCR reactions, performed at a total volume of 25 µL containing a PCR buffer (1×), a dNTP mix (0.2 mM), MgCl2 (1.5 mM), forward and reverse primers (0.5 µM each), Taq DNA polymerase (1 U), and 1 µL of genomic DNA. The reaction protocol comprised an initial denaturation at 94 °C for 3 min followed by 35 cycles of denaturation at 94 °C for 30 s, annealing at 55 °C (for the ITS region), 58 °C (for EF-1α), 54 °C (for GAPDH), or 62 °C (for OPA10-2) for 30 s, and an extension at 72 °C for 30 s, with a final extension at 72 °C for 10 min. The resulting amplicons were visualized on a 1% agarose gel, and the purified products were sequenced with both forward and reverse primers by Macrogen Europe (Amsterdam, The Netherlands). The sequences were analyzed using FinchTV v.1.4.0, and the obtained sequences were deposited in GenBank (Table 1). For molecular identification, the sequences from isolates characterized in this study and validated sequences of reference *Alternaria* isolates (Table 2) were used for phylogenetic analysis. Prior to analysis, the complete panel of reference sequences underwent the elimination of duplicates utilizing the Elim Dupes software (accessed on 15 June 2020). The sequences were aligned using MUSCLE and analyzed using MEGA6 for phylogenetic reconstruction using the maximum-likelihood method with the Tamura–Nei model [45]. Bootstrap analysis with 1000 replications was conducted. To enhance this investigation with the genetic diversity among the isolates obtained in this study, a combined dataset of all sequenced markers (ITS, EF-1α, GAPDH, and OPA10-2) was employed for phylogenetic analysis.

**Table 2:** GenBank accession numbers of sequences of *Alternaria* spp. isolates from different hosts and geographical origins used as references in phylogenetic analyses.

Species	Isolate	Country	Host	Source	Accession Numbers			
					ITS	EF-1α	GAPDH	OPA 10-2
*Alternaria* *alstroemeriae*	CBS 118808	USA	*Alstroemeria* sp.	[18]	KP124296	KP125071	KP124153	KP124601
*A. alternata*	AaMDc5b	Italy	*Punica granatum*	[4]	MW580731	MW585112	MW590490	MW590532
*A. alternata*	AaMDc5d	Italy	*P. granatum*	[4]	MW580755	MW585133	MW590514	MW590556
*A. alternata*	AaMMH6b	Italy	*P. granatum*	[4]	MW580756	MW585134	MW590515	MW590557
*A. alternata*	CBS 102.47	USA	*Citrus sinensis*	[18]	KP124304	KP125080	KP124161	KP124610
*A. alternata*	CBS 102595	USA	*C. jambhiri*	[18]	FJ266476	KC584666	AY562411	KP124636
*A. alternata*	CBS 112252	-	-	[18]	KP124340	KP125116	KP124194	KP124650
*A. alternata*	CBS 117.44	Denmark	*Godetia* sp.	[18]	KP124303	KP125079	KP124160	KP124609
*A. alternata*	CBS 916.96	India	*Arachis hypogaea*	[18]	AF347031	KC584634	AY278808	KP124632
*A. arborescens*	AaMDc1b	Italy	*P. granatum*	[4]	MW580737	MW585151	MW590496	MW590538
*A. arborescens*	AaMRa1	Italy	*P. granatum*	[4]	MW580759	MW585153	MW590518	MW590560
*A. arborescens*	CBS 108.41	-	wood	[18]	KP124394	KP125172	KP124246	KP124707
*A. arborescens*	CBS 109730	USA	*Solanum* *lycopersicum*	[18]	KP124399	KP125177	KP124251	KP124713
*A. arborescens*	CBS 112749	South Africa	*Malus domestica*	[18]	KP124401	KP125179	KP124253	KP124715
*A. arborescens*	CBS 115517	South Africa	*M. domestica*	[18]	KP124404	KP125182	KP124256	KP124718
*A. arborescens*	AaMDc1a	Italy	*P. granatum*	[4]	MW580736	MW585150	MW590495	MW590537
*A.* *betae-kenyensis*	CBS 118810	Kenya	*Beta vulgaris* var. *cicla*	[18]	KP124419	KP125197	KP124270	KP124733
*A. burnsii*	CBS 107.38	India	*Cuminum cyminum*	[18]	KP124420	JQ646305	KP125198	KP124734
*A. eichhorniae*	CBS 119778	Indonesia	*Eichhornia crassipes*	[18]	KP124426	KP125205	KP124277	KP124741
*A. jacinthicola*	CBS 878.95	Mauritius	*Arachis hypogaea*	[18]	KP124437	KP125216	KP124286	KP124753
*A. longipes*	CBS 540.94	USA	*Nicotiana tabacum*	[18]	AY278835	KC584667	AY278811	KP124758
*A. tomato*	CBS 103.30	-	*S. lycopersicum*	[18]	KP124445	KP125224	KP124294	KP124762

### 4.4. Pathogenicity Tests on Leaves and Fruits of Different Pomegranate Varieties

In order to assess the virulence of the *Alternaria* isolates and evaluate the susceptibility of the different pomegranate varieties, nine selected isolates representing all three diverse morphotypes and including both *A. alternata* (MNC1, MNC2, MNC3, MFO1, MFO2, and MF03) and *A. arborescens* (MFC1, MFC2, and MFC3) isolates were inoculated on the fruits and leaves of three pomegranate cultivars, ‘Acco’, ‘Etna’, and ‘Wonderful’. Fungal isolates were cultured on PDA in Petri dishes and incubated at 25 ± 1 °C for 7 days in the dark. A conidium suspension of each isolate was prepared by flooding the dishes with 6 mL of SDW and scraping the agar surface. The suspension was then filtered through a cheesecloth, and the concentration was adjusted to 1×10^6^ conidia/mL with SDW using a hemocytometer. In the pathogenicity tests, detached fully expanded leaves of the summer vegetative flushing and mature fruits of the three pomegranate cultivars were used. Both leaves and fruits were collected in November and, before inoculation, were surface disinfected by dipping them in 1% NaClO for 2 min, rinsed carefully with SDW, air-dried at room temperature, and then kept in humid chambers (plastic boxes with air-tight lids and damp paper on the bottoms) on aluminum foil or plastic rings, respectively, to avoid direct contact with the water. Artificial inoculations were performed by pipetting 20 µL drops of the conidial suspension on the abaxial side of the leaf lamina (four separate drops per leaf, two on each side of the midrib) and on the upper side of the fruit (six separate drops per fruit, in equatorial position). Mock inoculations were performed on the same number of leaves and fruits using sterile deionized water. The leaves and fruits were then incubated in humid chambers (plastic boxes) at 25 ± 1 °C under a 16/8 h light/dark photoperiod and 90% RH for 3 and 10 days, respectively. Three days after inoculation (dpi) on leaves and ten dpi on fruits, the diameter (*2r*) of the necrotic lesion at each inoculation site was measured, and the results were expressed as the area of the lesion calculated with the formula $A\;\left(\mathrm{area}\;\mathrm{of}\;\mathrm{circumference}\right)=\pi r^{2}$. To fulfil Koch’s postulates, reisolations were performed from symptomatic leaves and fruits using PDA amended with streptomycin sulfate as a medium. The isolates were identified on the basis of the colony morphology on PDA, and the identification of a restricted number of selected isolates was confirmed by sequencing ITS, 1-α (EF-1α), GAPDH, and OPA10–2. The pathogenicity test was repeated three times, each with 10 leaves and fruits per pomegranate cultivar and *Alternaria* isolate combination.

### 4.5. Statistical Analysis

The data from the pathogenicity tests were analyzed using RStudio v.4.3.1 (R). The means of the surface areas of the necrotic lesions induced by different *Alternaria* isolates on leaves and fruits were compared and analyzed by one-way analysis of variance (ANOVA) coupled with the Tukey–Kramer honestly significant difference (HSD) test.

## 5. Conclusions

This study presents the first report of Alternaria black spot of pomegranate in Italy, identifying *Alternaria alternata* and *A. arborescens* as the causal agents. The outbreak in Sicily followed an unusually rainy period, highlighting the role of environmental conditions in the disease’s emergence. Thirty *Alternaria* isolates from symptomatic pomegranate leaves and fruits were morphologically and molecularly characterized, revealing three distinct morphotypes. Morphotype 1 and 2 isolates, identified as *A. alternata*, were primarily found on fruits, while morphotype 3, identified as *A. arborescens*, was found on leaves. Pathogenicity tests showed all morphotypes could infect leaves of the cultivars ‘Acco’, ‘Wonderful’, and ‘Etna’, with ‘Acco’ being the least susceptible. Conversely, the fruit susceptibility varied: ‘Acco’ fruits were most susceptible to all morphotypes, whereas ‘Wonderful’ and ‘Etna’ fruits were only susceptible to morphotype 1 isolates. This study underscores the importance of cultivar selection and environmental monitoring in managing Alternaria black spot. The findings suggest the need for further research on cultivar resistance and pathogen variability, which are crucial for developing effective disease management strategies and breeding programs.

## Figures and Tables

**Table 1:** *Alternaria* isolates from fruits and leaves of pomegranate (*Punica granatum* L.) characterized in this study: colony morphology (morphotype), geographical origin and accession numbers of internal transcribed spacer (ITS), translation elongation factor 1-α (EF-1α), glyceraldehyde-3-phosphate dehydrogenase (GAPDH), and SCAR marker OPA 10-2 sequences in GenBank.

Isolate	Host, Cultivar	Source	Morphotype	Location	GenBank Accession Numbers			
					ITS	EF-1α	GPDH	OPA 10-2
MNC1	*P. granatum* ‘Wonderful’	Fruit	1	Sicily, IT	PP791893	PP820781	PP803578	PP803608
MNC2	*P. granatum* ‘Wonderful’	Fruit	1	Sicily, IT	PP791895	PP820783	PP803580	PP803610
MNC3	*P. granatum* ‘Wonderful’	Fruit	1	Sicily, IT	PP791894	PP820782	PP803579	PP803609
MNC4	*P. granatum* ‘Wonderful’	Fruit	1	Sicily, IT	PP791896	PP820784	PP803581	PP803611
MNC5	*P. granatum* ‘Wonderful’	Fruit	1	Sicily, IT	PP791897	PP820785	PP803582	PP803612
MNC6	*P. granatum* ‘Wonderful’	Fruit	1	Sicily, IT	PP791898	PP820786	PP803583	PP803613
MNC7	*P. granatum* ‘Wonderful’	Fruit	1	Sicily, IT	PP791899	PP820787	PP803584	PP803614
MNC8	*P. granatum* ‘Wonderful’	Fruit	1	Sicily, IT	PP791900	PP820788	PP803585	PP803615
MNC9	*P. granatum* ‘Wonderful’	Fruit	1	Sicily, IT	PP791901	PP820789	PP803586	PP803616
MNC10	*P. granatum* ‘Wonderful’	Fruit	1	Sicily, IT	PP791902	PP820790	PP803587	PP803617
MFO1	*P. granatum* ‘Wonderful’	Leaf	2	Sicily, IT	PP791890	PP820778	PP803575	PP803605
MFO2	*P. granatum* ‘Wonderful’	Leaf	2	Sicily, IT	PP791889	PP820777	PP803574	PP803604
MFO3	*P. granatum* ‘Wonderful’	Leaf	2	Sicily, IT	PP791888	PP820776	PP803573	PP803603
MFO4	*P. granatum* ‘Wonderful’	Leaf	2	Sicily, IT	PP791887	PP820775	PP803572	PP803602
MFO5	*P. granatum* ‘Wonderful’	Leaf	2	Sicily, IT	PP791886	PP820774	PP803571	PP803601
MFO6	*P. granatum* ‘Wonderful’	Leaf	2	Sicily, IT	PP791885	PP820773	PP803570	PP803600
MFO7	*P. granatum* ‘Wonderful’	Leaf	2	Sicily, IT	PP791883	PP820771	PP803568	PP803598
MFO8	*P. granatum* ‘Wonderful’	Leaf	2	Sicily, IT	PP791884	PP820772	PP803569	PP803599
MFO9	*P. granatum* ‘Wonderful’	Leaf	2	Sicily, IT	PP791891	PP820779	PP803576	PP803606
MFO10	*P. granatum* ‘Wonderful’	Leaf	2	Sicily, IT	PP791892	PP820780	PP803577	PP803607
MFC1	*P. granatum* ‘Wonderful’	Leaf	3	Sicily, IT	PP791903	PP820791	PP803588	PP803618
MFC2	*P. granatum* ‘Wonderful’	Leaf	3	Sicily, IT	PP791904	PP820792	PP803589	PP803619
MFC3	*P. granatum* ‘Wonderful’	Leaf	3	Sicily, IT	PP791905	PP820793	PP803590	PP803620
MFC4	*P. granatum* ‘Wonderful’	Leaf	3	Sicily, IT	PP791906	PP820794	PP803591	PP803621
MFC5	*P. granatum* ‘Wonderful’	Leaf	3	Sicily, IT	PP791907	PP820795	PP803592	PP803622
MFC6	*P. granatum* ‘Wonderful’	Leaf	3	Sicily, IT	PP791908	PP820796	PP803593	PP803623
MFC7	*P. granatum* ‘Wonderful’	Leaf	3	Sicily, IT	PP791909	PP820797	PP803594	PP803624
MFC8	*P. granatum* ‘Wonderful’	Leaf	3	Sicily, IT	PP791910	PP820798	PP803595	PP803625
MFC9	*P. granatum* ‘Wonderful’	Leaf	3	Sicily, IT	PP791911	PP820799	PP803596	PP803626
MFC10	*P. granatum* ‘Wonderful’	Leaf	3	Sicily, IT	PP791912	PP820800	PP803597	PP803627

## Data Availability

Data is contained within the article.
